# Antitumor effect of D-erythrose in an abdominal metastatic model of colon carcinoma

**DOI:** 10.3892/ol.2014.2764

**Published:** 2014-12-03

**Authors:** LI-LI LIU, TAO YI, XIA ZHAO

**Affiliations:** Department of Gynecology and Obstetrics, Key Laboratory of Obstetric, Gynecological and Pediatric Diseases and Birth Defects of Ministry of Education, West China Second Hospital, Sichuan University, Chengdu, Sichuan 610041, P.R. China

**Keywords:** D-erythrose, colon cancer, Warburg effect, apoptosis

## Abstract

Traditional chemotherapy drugs against colorectal cancer possess little or no specificity, leading to severe intolerable side-effects. Therefore, it is necessary to develop additional specific therapeutic strategies. It has been suggested that D-erythrose may specifically inhibit the growth of tumor cells. However, the *in vivo* antitumor effect of D-erythrose against colorectal cancer remains unknown. Thus, the present study investigated the antitumor effect of D-erythrose in an abdominal metastatic model of colon carcinoma. Intraperitoneal (IP) colon carcinoma-bearing BALB/c mice received an IP injection of D-erythrose or normal saline (NS) daily for 15 days. The mice were weighed every three days. The tumor weights and the volume of ascites were evaluated following the treatment. Terminal deoxynucleotidyl transferase-mediated dUTP nick end labeling assay was used to assess apoptosis in tumor tissues. The results revealed that D-erythrose significantly reduced the weight of the intraperitoneal tumor by 69.1%, markedly inhibited the development of ascites and increased tumor cell apoptosis, without any observed toxic effects. These observations suggest that D-erythrose possesses antitumor activity against colon cancer. The present study may provide a potentially effective and specific approach for colon cancer treatment.

## Introduction

Colorectal cancer remains the leading cause of cancer-associated death in developed and developing countries (1). Despite progress in surgery, chemotherapy, biotherapy and radiotherapy, the mortality rate of colorectal cancer remains high. In total, ~136,830 novel cases and 50,310 mortalities are estimated to occur due to colorectal cancer in the United States in 2014 (2). Lack of specificity is one of the main factors that limit the efficacy of treatment, which leads to severe intolerable side-effects (3). Therefore, the development of effective and tumor-specific therapeutic approaches is urgently required.

Previous studies have suggested that the unique metabolism of cancer cells may become a novel target of tumor-specific therapy (4–8). Cancer cells possess a different bioenergetic metabolism to that of normal cells. Normal cells mostly depend on mitochondrial oxidative phosphorylation to produce energy, while cancer cells primarily depend on glycolysis, even in the presence of oxygen. This altered metabolism is known as the Warburg effect and gives rise to enhanced lactate production (9–13). Targeting this type of metabolism may offer a possibility for tumor-specific treatment.

D-erythrose is a tetrose carbohydrate that exists in the human body in the form of D-erythrose 4-phosphate, which is an intermediate in the pentose phosphate pathway (14). The metabolism of administered D-erythrose in the human body remains unknown. Although the transformation between D-erythrose and D-erythrose 4-phosphate occurs in certain microorganisms (15), there are no studies investigating this transformation in the human body. A previous study using radioactive D-erythrose revealed that D-erythrose could be utilized by rats, as the radioactivity eventually appeared in carbon dioxide and glucose, indicating that the administered D-erythrose can be completely oxidized to carbon dioxide (16). Based on the aforementioned findings, at the 101st Annual Meeting of the American Association for Cancer Research, Wang and Wei (17) put forward the hypothesis that, in tumor tissues, the administered D-erythrose is oxidized in the cytosol or mitochondria to carbon dioxide, which is then converted to carbonic acid under the catalysis of carbonic anhydrase. Since cancer cells possess increased lactate production, this results in increased formation of lactic acid and intracellular acidosis, which can ultimately cause cell death once the acidosis exceeds a certain threshold. In the previous study by Wang and Wei, D-erythrose was used as an antitumor agent, and it was found that D-erythrose could inhibit the viability of several cancer cell lines *in vitro*, and suppress the growth of lung cancer in a subcutaneous tumor model of LL-2 Lewis lung cells *in vivo*.

However, little is known about the antitumor activity of D-erythrose in colorectal cancer in an animal model. Therefore, in the present study, the antitumor effect of D-erythrose in an abdominal metastatic model of colon carcinoma was investigated. The present results revealed that intraperitoneal administration of D-erythrose significantly suppressed the growth of colon carcinoma, reduced the development of ascites and increased tumor cell apoptosis.

## Materials and methods

### Cell culture

The mouse colon adenocarcinoma C-26 cell line (American Type Culture Collection, Manassas, VA, USA) was cultured in RPMI-1640 medium supplemented with 10% fetal bovine serum. The cells were incubated at 37°C in a 5% CO_2_ humidified incubator and passaged every three days using trypsin.

### Establishment of the animal model and therapy studies

The following procedures were approved by the Institutional Animal Care and Use Committee of Sichuan University (Chengdu, China). To evaluate the antitumor effect of D-erythrose in colon cancer *in vivo*, an abdominal metastatic model of colon carcinoma was established, as previously described (18). Male BALB/c mice (eight to nine weeks old) were obtained from the Laboratory Animal Center of Sichuan University and received IP injections of 0.2 ml C-26 cell suspension, containing 2×10^5^ cells. Three days after the injection (day 0), the mice were randomly divided into two groups, with eight mice per group, and received daily IP administration of D-erythrose (500 mg/kg; Carbosynth, Compton, UK) or equal volume of normal saline (NS), respectively, for the following 15 days. The weight of the mice was recorded every three days. All mice were sacrificed by cervical dislocation 24 h after the last administration and the tumors were collected and weighed. The tumor inhibition rate was calculated by the following formula: Tumor inhibition rate (%) = (1 − mean tumor weight in D-erythrose-treated group / mean tumor weight in NS group) × 100. The volume of ascites was recorded at the same time.

### Histological analysis

Tumors of each group were fixed in 10% formalin (pH 7.0) for ≥24 h, and then embedded in paraffin. The paraffin-embedded tumors were then sliced into 3–5 μm sections. Subsequent to slicing, a terminal deoxynucleotidyl transferase-mediated dUTP nick end labeling (TUNEL) assay was performed to detect apoptotic cells in the tumor tissues using the DeadEnd^TM^ Fluorometric TUNEL System, according to the manufacturer’s instructions (Promega, Madison, WI, USA). The cell nuclei stained with green fluorescence were identified as TUNEL-positive nuclei. The apoptosis index was calculated by analyzing the average percentage of TUNEL-positive cells in five random fields from at least three different sections at a magnification of ×400.

### Toxicity assessment

To evaluate the possible side effects of D-erythrose, health-associated indexes, including anorexia, diarrhea, skin ulceration and toxic death, were observed every three days. Furthermore, the main organs of the mice, including the heart, liver, spleen, lung and kidney, were collected for hematoxylin and eosin (H&E) staining.

### Statistical analysis

The data were recorded as the mean ± standard error. The two-tailed unpaired Student’s t-test was used for comparison. P<0.05 was considered to indicate a statistically significant difference.

## Results

### Antitumor effect of D-erythrose in an abdominal metastatic model of colon carcinoma

**Figure 1:**
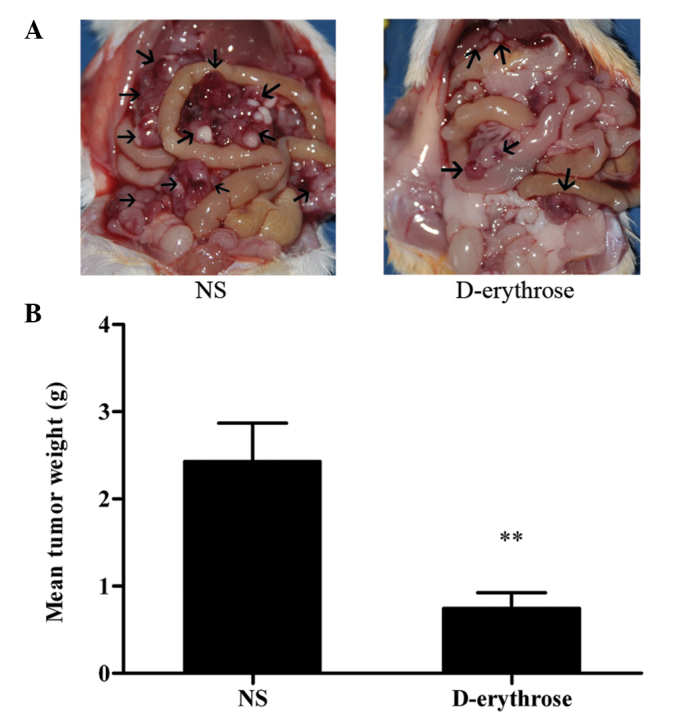
Intraperitoneal administration of D-erythrose inhibited the growth of intraperitoneal metastases of C-26 colon carcinoma. (A) Representative images of the intraperitoneal metastases of C-26 colon carcinoma in each group. (B) Weights of intraperitoneal metastases in each group. Data are expressed as mean ± standard error. ^**^P<0.01, vs. the NS group. NS, normal saline.

The abdominal metastatic model of colon carcinoma was used to evaluate the antitumor effect of D-erythrose in colon cancer. The mice were administered daily with D-erythrose (500 mg/kg) or NS, respectively, for 15 days. All mice were sacrificed 24 h after the last treatment. Fig. 1A shows representative images of intraperitoneal metastases of C-26 colon carcinoma in the two groups. It is clear that mice treated with D-erythrose bore fewer intraperitoneal metastases than mice treated with NS. The metastases in each group were collected and weighed. As shown in Fig. 1B, the mean tumor weight was 0.75±0.17 in the D-erythrose-treated group vs. 2.43±0.44 g in the NS group. IP administration of D-erythrose (500 mg/kg) significantly suppressed tumor growth compared with the control agent, with a tumor inhibition rate of 69.1% (P<0.01).

### D-erythrose reduces the development of ascites

**Figure 2:**
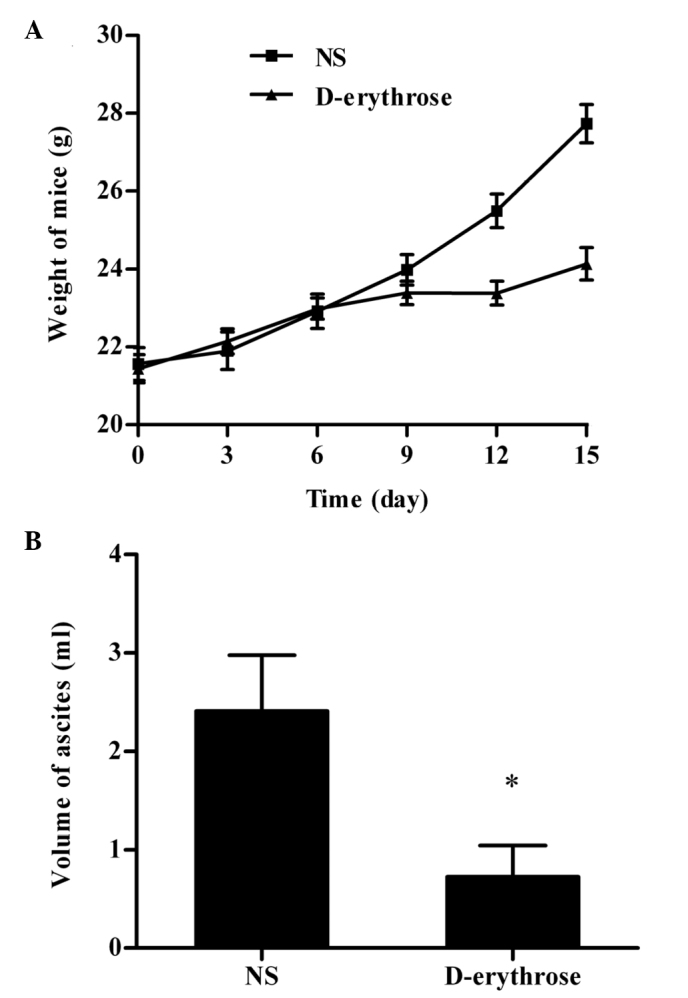
Intraperitoneal administration of D-erythrose reduced the development of ascites. (A) Effect of D-erythrose on ascites-associated weight gain. The weight of mice was recorded every three days. The late rapid weight gain in the NS group reflects the accumulation of ascites. The ascites-associated weight gain was evidently reduced in the D-erythrose-treated group. (B) Volume of ascites in each group. The mice that were sacrificed 24 h after withdrawal of D-erythrose possessed smaller ascites volumes compared with the controls. The data are presented as the mean ± standard error. ^*^P<0.05, vs. the NS group. NS, normal saline.

The mice were weighed every three days and the weights of the mice increased rapidly over time due to the accumulation of ascites. As shown in Fig. 2A, the ascites-associated weight gain was evidently slower in the D-erythrose-treated group.

The volume of ascites was recorded subsequent to the mice being sacrificed (Fig. 2B). In the NS group, hemorrhagic ascites developed in seven of the eight mice. By contrast, hemorrhagic ascites only developed in four of the eight mice in the D-erythrose-treated group. In addition, the mean volume of ascites in the D-erythrose-treated group was significantly smaller compared with that of the NS group (P<0.05).

### D-erythrose increases apoptosis in tumor tissues

**Figure 3:**
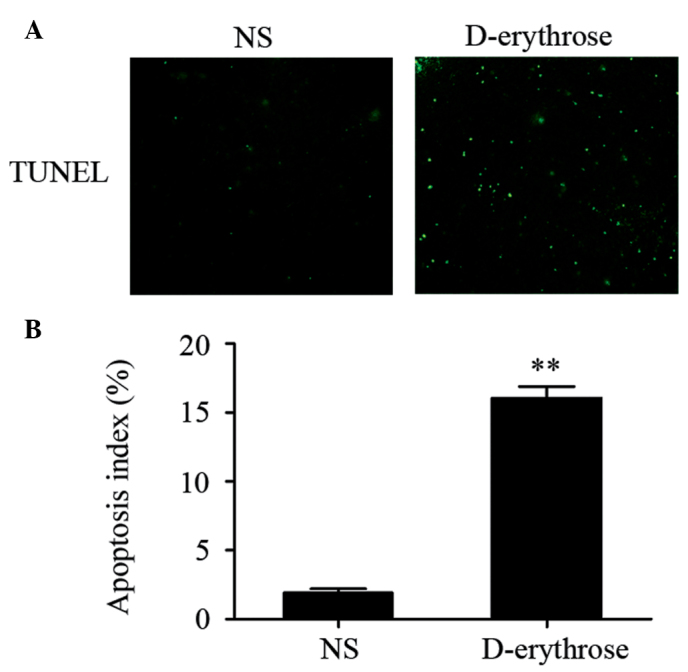
TUNEL staining of tumor sections in each group. (A) Tumor sections stained for TUNEL (magnification, ×200). Numerous TUNEL-positive cells were observed in the D-erythrose treated group, whereas such cells were rare in the NS group. (B) Quantification of TUNEL staining (apoptotic index). The data are expressed as the mean ± standard error. ^**^P<0.01, vs. the NS group. TUNEL, terminal deoxynucleotidyl transferase-mediated dUTP nick end labeling; NS, normal saline.

A TUNEL assay was used to detect apoptotic tumor cells in each group. The cell nuclei stained with green fluorescence were considered to be TUNEL-positive nuclei. As shown in Fig. 3, the percentage of TUNEL-positive cells (apoptotic cells) was significantly increased in the D-erythrose treated group compared with that in the NS group (P<0.01). Apoptotic cells were rare in the NS group.

### Toxicity observation

No evident abnormalities, including anorexia, diarrhea, skin ulceration and toxic death, were noted in the two groups. Additionally, H&E staining of the heart, liver, spleen, lung and kidney did not exhibit any clear pathological alteration in the D-erythrose treated group compared with the NS group.

## Discussion

The antitumor effect of D-erythrose in an abdominal metastatic model of colon carcinoma was observed in the present study. The results revealed that IP administration of D-erythrose significantly suppressed the growth of intraperitoneal tumors compared with the control agent, as revealed by mean tumor weight analysis (Fig. 1). The present results are similar to those obtained by Wang and Wei, who subcutaneously injected D-erythrose beside a tumor in a subcutaneous tumor model of LL-2 Lewis lung cells (17). Furthermore, in the present study, malignant ascites were markedly reduced in the D-erythrose-treated group (Fig. 2), indicating that D-erythrose may also inhibit tumor invasion. The present study provides proof of the principle that D-erythrose possesses antitumor activity against colon cancer, and provides a focus for future investigations.

The inhibition of apoptosis is considered essential for tumor growth, and therefore induction of tumor cell apoptosis has been demonstrated to be a promising strategy for tumor therapy (19,20). In the present study, TUNEL staining of the tumor tissue sections demonstrated that IP administration of D-erythrose resulted in increased apoptosis of colon cancer cells compared with the cells treated with the control agent (Fig. 3), indicating that D-erythrose may exert its antitumor effect by induction of apoptosis.

Toxicity is an important factor that influences the ultimate therapeutic effect of antitumor drugs (21). In the present study, no gross abnormalities, including anorexia, diarrhea, skin ulceration and toxic death, were noticed during the *in vivo* treatment period. In addition, H&E staining of the major organs (heart, liver, spleen, lung and kidney) did not exhibit any evident pathological alteration following treatment with D-erythrose. These results indicate that the administration of D-erythrose appears to be safe and without detectable systemic toxic effects, at least at the dose used (500 mg/kg). Furthermore, according to the public report by the National Industrial Chemicals Notification and Assessment Scheme, erythrulose, an isomer of erythrose, exhibits low acute oral toxicity in rats (acute oral mean lethal dose, >2 g/kg), and the no-observed-effect-level was 1 g/kg/day in a 28-day repeat dose oral toxicity study (22). The aforementioned results reveal that the administration of D-erythrose (500 mg/kg) exhibits a low toxicity and possesses potential clinical applications.

The antitumor mechanism of D-erythrose may be associated with the unique bioenergetic metabolism of cancer cells. Differing from normal cells, cancer cells mostly depend on glycolysis rather than mitochondrial oxidative phosphorylation to produce energy, even in the presence of ample oxygen. This phenomenon was first reported by the Nobel Prize winner Warburg 90 years ago (9), and has repeatedly been observed in various types of cancer cells. The increased dependency upon glycolysis is a hallmark of cancer cell metabolism, and gives rise to enhanced lactate production (11,12). D-erythrose, a tetrose carbohydrate, can be used as cellular fuel, and its final products are carbon dioxide and water (16). According to the hypothesis of Wang and Wei, in cancer tissues, D-erythrose is oxidized to carbon dioxide, which is then converted to carbonic acid catalyzed by carbonic anhydrase (17). Due to the increased lactate production in cancer cells, it ultimately results in an increased formation of lactic acid. The lactic acid-induced acidosis, once above a certain threshold, can finally lead to cancer cell death. However, the exact mechanism requires further investigation.

One of the key factors that restricts therapeutic advances is the lack of tumor-specific therapy. Traditional chemotherapy drugs against colorectal cancer, including 5-fluorouracil, irinotecan and oxaliplatin, are widely used in clinical practice. However, they exhibit little or no specificity, leading to various side effects that necessitate a dose reduction or even termination of the therapy (23–27), and any extremely serious side effect impairs the quality of life of the individual. Therefore, it is necessary to develop additional specific therapeutic strategies. For this purpose, the unique bioenergetic metabolism of cancer cells has been increasingly investigated (5–7). Based on the high levels of glycolysis and lactate production in cancer cells, D-erythrose significantly inhibited the growth of colon cancer *in vivo* in the present study, without any observed effect on normal tissues, indicating that D-erythrose may become a potentially effective and specific antitumor agent against colon cancer. In addition, D-erythrose may be more effective in combination with other antitumor drugs, which provides an attractive therapeutic strategy for further investigation.

In conclusion, the present data suggest that D-erythrose can markedly suppress the growth of colon carcinoma, inhibit tumor cell invasion and increase tumor cell apoptosis, without any observed toxic effects. The present study may provide an effective and specific therapeutic strategy for colon cancer treatment.
